# Tonotopic organization of the hyperpolarization-activated current (I_h_) in the mammalian medial superior olive

**DOI:** 10.3389/fncir.2013.00117

**Published:** 2013-07-11

**Authors:** Veronika J. Baumann, Simon Lehnert, Christian Leibold, Ursula Koch

**Affiliations:** ^1^Division of Neurobiology, Department Biology II, Ludwig-Maximilians-Universität MünchenMunich, Germany; ^2^Institute of Biology, Fachbereich Biologie, Chemie, Pharmazie, Freie Universität BerlinBerlin, Germany

**Keywords:** HCN channel, medial superior olive, sound localization, tonotopy, coincidence detection

## Abstract

Neuronal membrane properties can largely vary even within distinct morphological cell classes. The mechanisms and functional consequences of this diversity, however, are little explored. In the medial superior olive (MSO), a brainstem nucleus that performs binaural coincidence detection, membrane properties at rest are largely governed by the hyperpolarization-activated inward current (I_h_) which enables the temporally precise integration of excitatory and inhibitory inputs. Here, we report that I_h_ density varies along the putative tonotopic axis of the MSO with I_h_ being largest in ventral, high-frequency (HF) processing neurons. Also I_h_ half-maximal activation voltage and time constant are differentially distributed such that I_h_ of the putative HF processing neurons activate faster and at more depolarized levels. Intracellular application of saturating concentrations of cyclic AMP removed the regional difference in hyperpolarization-activated cyclic nucleotide gated (HCN) channel activation, but not I_h_ density. Experimental data in conjunction with a computational model suggest that increased I_h_ levels are helpful in counteracting temporal summation of phase-locked inhibitory inputs which is particularly prominent in HF neurons.

## Introduction

Neuronal encoding of information in the time domain is enhanced by specific adjustments of membrane properties to the dynamics and temporal characteristics of the inputs (O'Donnell and Nolan, 2011). This is especially important for neurons in the medial superior olive (MSO), a binaural nucleus in the auditory brainstem that analyses interaural time differences (ITDs) of different input frequencies with extremely high temporal precision. This acuity primarily relies on the coincidence detection of precisely timed excitatory inputs from both ears onto MSO neurons (Grothe et al., 2010). In addition, two glycinergic inputs, originating from the ipsilateral medial and lateral nucleus of the trapezoid body, provide a prominent and phase-locked inhibition to MSO neurons, which fine-tunes the slope of the ITD function to occur within the physiological range (Brand et al., 2002; Pecka et al., 2008; Leibold, 2010). Equally important for the high temporal precision with which these neurons integrate their excitatory and inhibitory inputs are the large voltage-gated channels that are open around the resting potential of the membrane. Such exquisitely fine-tuned temporal processing crucially depends on the composition and the properties of voltage-gated ion channels. One of these voltage-gated currents is I_h_ (or HCN-current), a cationic current, which is activated upon hyperpolarization (Wahl-Schott and Biel, 2009). I_h_ is especially large in MSO neurons and is regulated by intrinsic modulators such as cAMP and PIP2 (Khurana et al., 2012). In addition, these neurons also express a large low voltage-activated K^+^-channel (K_LVA_) that also opens around the resting potential (Barnes-Davies et al., 2004; Mathews et al., 2010; Khurana et al., 2011). The sophisticated interplay between these channels reduces the input resistance and shortens the membrane time constant and thereby enhances the temporal acuity with which these neurons integrate their synaptic inputs (Barnes-Davies et al., 2004; Hassfurth et al., 2009; Mathews et al., 2010; Karcz et al., 2011; Khurana et al., 2011).

Like most nuclei in the auditory brainstem, the MSO is tonotopically organized: Low-frequency (LF) sounds are represented dorsally and higher frequencies are processed ventrally (Guinan et al., 1972; Müller, 1990). This spatial gradient of input frequencies enabled us to investigate the relationship between I_h_ properties, the integration of inhibitory inputs and its dependence on input frequency in the acute brain slice preparation using whole-cell patch-clamp recordings. We found that I_h_ is differentially distributed along the dorsoventral axis of the nucleus and that this spatial arrangement is paralleled by differential properties of synaptic integration.

Moreover, we explored the putative functional consequences of this relationship theoretically using a computational single-compartment model featuring HCN and K_LVA_ channels that was fitted to electrophysiological recordings: this model suggests that integration of inhibitory inputs in a frequency-dependent manner helps to maintain the neuron's membrane potential close to firing threshold.

## Materials and methods

All experiments were performed in accordance with the rules laid down by the EC Council Directive (86/89/ECC) and German animal welfare legislation and approved by the Regierung Oberbayern (AZ 55.2-1-54-2531-57-05, Bavaria, Germany). All agents were purchased from Sigma-Aldrich (Germany) and Biotrend (Germany) unless otherwise indicated.

### Slice preparation

Patch-clamp recordings were performed from MSO neurons of gerbils (*Meriones unguiculatus*) at the age of postnatal day 17/18 (denoted P18) and 21/22/23 (denoted P22). The animals were decapitated under isoflurane anesthesia. The brain was removed in ice-cold oxygenated (95% O_2_/5% CO_2_) sucrose replacement solution containing (in mM): 2.5 KCl, 1.25 NaH_2_PO_4_, 26 NaHCO_3_, 0.25 CaCl_2_, 3 MgCl_2_, 12.5 glucose and 100 sucrose (pH 7.4). Transverse brainstem slices (180 μm) comprising the MSO were cut with a vibratome (VT1200S; Leica, Germany), incubated at 32°C for 15 min in oxygenated artificial cerebrospinal fluid (ACSF) containing (in mM) 125 NaCl, 2.5 KCl, 1.25 NaH_2_PO_4_, 26 NaHCO_3_, 2 CaCl_2_, 1 MgCl_2_ and 25 glucose and then maintained at room temperature. For recordings, slices were transferred to a recording chamber, which was perfused continuously with oxygenated ACSF at 32°C, and visualized with an upright microscope (Axioscope, Zeiss, Germany) using infrared-differential interference contrast optics.

### Electrophysiology

Current- and voltage-clamp recordings were made from visually identified MSO cells using a Multiclamp 700 A amplifier (Axon Instruments, USA) with standard electrode solution containing (in mM): 125 K-gluconate, 5 KCl, 10 HEPES, 1 EGTA, 2 Na_2_ATP, 2 MgATP, 0.3 Na_2_GTP and 10 Na-phosphocreatine; adjusted to pH 7.25 with KOH. All experiments were performed at near-physiological temperature (32°C). Patch pipettes were pulled from borosilicate glass capillaries (BioMedical Instruments, Germany) on a DMZ Universal Puller (Zeitz Instruments, Germany). When filled with electrode solution, patch pipettes had a resistance of 2–4 MΩ. In some experiments Alexa-488 (100 μM) (Molecular Probes, Germany) was added to the electrode solution in order to verify the location of the neuron along the presumed tonotopic axis.

During voltage-clamp recordings, whole-cell capacitance was compensated and used as measure for cell surface. The series resistance (<10 MΩ) was compensated to a residual of 2–2.5 MΩ and not allowed to change more than 20%. To isolate I_h_ pharmacologically we applied the following drugs (in mM): 1 3,4 diaminopyridine, 10 TEA-Cl, 0.2 BaCl_2_, 0.001 TTX, 0.05 NiCl_2_, 0.1 CdCl_2_, 0.01 DNQX, 0.025 DL-AP5 and 0.001 strychnine. NaCl was reduced to maintain iso-osmolarity.

We cannot exclude that our voltage-clamp recordings are distorted due to space-clamp errors which result in incomplete control of dendritic membrane potential. We minimize these errors by using an I_h_ isolation cocktail. Additionally, MSO neurons are anatomically compact cells with short dendrites (~150 μm) (Rautenberg et al., 2009) so that space-clamp errors should be small. Moreover, it is likely that the somatic voltage-clamp underestimates the HCN channel conductance.

During current-clamp experiments, the bridge-balance was adjusted to compensate for artifacts arising from electrode resistance. In some experiments, I_h_ was blocked with the HCN channel-selective inhibitor ZD7288 (20 μM).

Synaptic currents were evoked stimulating the slice with a glass electrode filled with 2 M NaCl. Stimulation electrodes were placed medial and lateral to the MSO. Inhibitory postsynaptic currents (IPSCs) were isolated by addition of 10 μM DNQX and 25 μM DL-AP5. IPSCs were evoked by brief pulses (100 μs, intensities 10–40 V) triggered by an analogue stimulus isolation unit (BSI-950, Dagan Corporation, USA). Patch electrodes were filled with (in mM) 99 CsMeSO_4_, 41 CsCl, 10 HEPES, 10 EGTA, 2 Na_2_ATP, 2 MgATP, 0.3 Na_2_GTP, 5 TEA-Cl and 1 CaCl_2_, and 5 QX314 to block postsynaptic Na^+^ channels; adjusted to pH 7.25 with CsOH.

In conductance-clamp experiments, simulated inhibitory conductances at 100 Hz were injected into MSO neurons with a SM-1 amplifier (Cambridge Conductance, UK). The simulated inhibitory conductance based upon recorded IPSCs (decay time: ~1.5 ms, 10–90% rise time: ~0.9 ms, reversal potential: −90 mV). The reversal potential was chosen according to data by Magnusson et al. (2005).

### Data acquisition and analysis

Both voltage and current signals were low-pass filtered at 10 kHz with a four-pole Bessel filter and sampled at a rate of 20–50 kHz. Stimulus generation and recordings were done with pCLAMP (Axon Instruments, USA). All electrophysiological data were analysed in IGOR Pro (Wavemetrics, USA) using Neuromatic and custom-written routines, or in Clampfit (Axon Instruments, USA). A junction potential of −10.5 mV was corrected.

Steady-state current responses were evaluated at the end of the voltage pulse. I_h_ density was obtained by normalizing the amplitude to the compensated whole-cell capacitance. The voltage dependence of I_h_ activation was measured from the tail current. Values were fitted with a Boltzmann function to obtain the half-maximal activation voltage *V*_0.5_: *f*(*V*) = 1/(1 + exp [(*V*_0.5_ − *V/k*)]), where *V* is the membrane voltage and *k* is the slope factor. The membrane time constants were evaluated by fitting a double-exponential function to the current traces: *f*(*t*) = *A*_1_ exp (−*t*/τ_fast_) + *A*_2_ exp (−*t*/τ_slow_) where τ_fast_ and τ_slow_ are the fast and slow time constant of I_h_ activation. The effective time constant of I_h_ activation, τ_weighted_, was calculated according to: τ_weighted_ = (*A*_1_^*^τ_fast_ + *A*_2_^*^τ_slow_)/(*A*_1_ + *A*_2_). *V*_0.5_ and τ_weighted_ were estimated for each experiment and averaged.

Input resistance was assessed from the peak hyperpolarization triggered by −100 pA current injection according to Ohm's law *R* = *U/I*. The membrane time constant was estimated from a single-exponential fit to the voltage response to −100 pA current injection.

To determine decay times of evoked IPSCs the decay was fitted with a single-exponential function. The time course of inhibitory postsynaptic potentials (IPSPs) was analyzed by averaging 30 traces, normalizing the resulting trace to the first IPSP amplitude, and then the 10–90% rise time, the 90–10% decay time and the half-width of the IPSPs were estimated.

Results are expressed as mean ± standard error of the mean (SEM). Statistical significance was determined by a single-factor ANOVA test followed by a Scheffé's *post-hoc* test or by Student's unpaired *t*-test in Excel (Microsoft) with significance thresholds of *P* < 0.05 (^*^), *P* < 0.01 (^**^), and *P* < 0.001 (^***^).

### Reconstruction of patched neurons

Following recording, slices were fixed in 4% paraformaldehyde for 30 min. After extensive washing in phosphate-buffered saline (PBS) slices were exposed to blocking buffer (0.5% trition X-100/0.1% saponin/1% BSA in PBS) followed by incubation with the primary antibody (chicken anti-microtubule-associated protein 2, MAP2, 1:1000, Neuromics) in blocking buffer. Slices were then rinsed in washing buffer (0.5% Trition X-100/0.1% saponin in PBS) and immunoreactivity was visualized by incubating the slices with the Cy3-conjugated secondary antibody raised in donkey (1:300; Dianova). Finally, slices were washed and mounted on slides with vectashield mounting reagent (Vector Laboratories, USA).

### Modeling

A Hodgkin-Huxley-type single-compartment model was implemented separately for prototypic P22 dorsal and ventral cells. The temporal evolution of membrane potential *V* followed the differential equation

$$C_{m}\frac{dV}{dt}=-\left(I_{h}+I_{\text{KLT}}+I_{\text{syn}}+I_{\text{leak}}\right)$$

with membrane capacitance *C*_*m*_ and Ohmic currents

$$I_{x}(V)=g_{x}a_{x}^{m}b_{x}^{n}(V-E_{x}).$$

The parameter *g*_*x*_ describes the peak conductance, *a*_*x*_ and *b*_*x*_ are the gating variables for activation and inactivation, respectively, and *E*_*x*_ denotes the reversal potential. The gating variables follow first order kinetics

$$\frac{da}{dt}=\frac{a_{\infty }-a}{\tau_{a}}\text{ and }\frac{db}{dt}=\frac{b_{\infty }-b}{\tau_{b}}$$

with the steady-state activation *a*/*b*_∞_ and the voltage-dependent time constants τ_*a*/*b*_.

The low-threshold potassium channel (KLT) was modeled according to Mathews et al. (2010) with *E*_*K*_ = −90 mV. The kinetics of the hyperpolarization-activated cation current (I_h_) was fitted to the data of voltage-clamp experiments from Figure 5, which resulted in the steady-state activation and the activation time constant (see Figure 7A)

$$a_{\infty }(V)={\left(1+e^{0.1(V\;+\;80.4)}\right)}^{-1}\text{ and }\tau_{a}=79+417e^{-{\left(V\;+\;61.5\right)}^{2}/800}$$

for dorsal cells, and

$$a_{\infty }(V)={\left(1+e^{0.095(V\;+\;75.5)}\right)}^{-1}\text{ and }\tau_{a}=65+292e^{-{\left(V\;+\;62.5\right)}^{2}/722}.$$

for ventral cells, respectively (*V* in mV). Since HCN channels do not spontaneously inactivate, *b* was set to 1. As reversal potential we used *E*_*h*_ = −35 mV.

The model has been adapted to the different mean values of the membrane properties of the ventral (HF) and dorsal (LF) population by using the following channel peak conductances (in nS/μ m^2^): g_KLT^dorsal^_ = 0.0531, g_HCN^dorsal^_ = 0.01025, g_KLT^ventral^_ = g_KLT^dorsal^_^*^ 5.4 and g_HCN^ventral^_ = g_HCN^dorsal^_^*^ 3.15. These settings yield a resting potential of around −60 mV for both model types and input resistances of *R*_in_ = 23.94 MΩ for the dorsal and *R*_in_ = 3.77 MΩ for the ventral model corresponding to membrane time constants of τ_*m*_ of 1.64 ms and 0.45 ms, respectively. Using a specific membrane capacitance of 1 μF/cm^2^ these correspond to a modeled cell surface of 6839 μm^2^ (dorsal) and 12064 μm^2^ (ventral) with membrane capacities of 68.39 pF and 120.64 pF, respectively.

For both cell models the passive leak conductance was set to g_leak_ = 33.3 fS/μm^2^ and the reversal potential was set to −70 mV.

The fitting procedure described above implicates that the model parameters (specifically the HCN conductances) are adjusted according to our current-clamp data. This was done on purpose, since we assume the voltage-clamp data to be less accurate due to the above mentioned incomplete voltage-clamp control especially in the dendrites.

The inhibitory input to the model was implemented as a conductance with reversal potential of −90 mV. The IPSG kinetics were fitted with a double-exponential (*t* in ms) to resemble measurements from Couchman et al. (2010):

$$G(t)=g_{\text{inh}}\frac{\left(1-e^{-t/0.4}\right)e^{-t/1.6}}{\max\left(\left(1-e^{-t/0.4}\right)e^{-t/1.6}\right)}$$

For simulations to investigate the IPSP half-widths inhibitory 100 Hz input stimuli of 20.5 nS (dorsal) and 90 nS (ventral) were applied to the models to roughly fit the membrane potential deflection seen in the corresponding current clamp experiments. The stimulus train was kept up for 800 ms to show the influence of the slowly activating HCN current.

## Results

### I_H_ varies along the dorsoventral axis in the MSO

**Figure 1:**
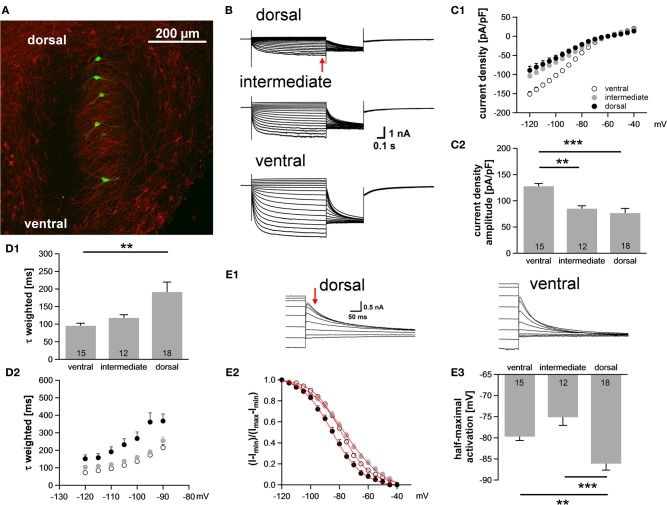
**I_h_ varies systematically along the dorsoventral axis. (A)** A brain slice containing the MSO with Alexa-488-filled neurons (green) verifies the distribution of the patched neurons along the dorsoventral axis (red: MAP-2). **(B)** Pharmacologically isolated I_h_ current traces were elicited by depolarizing and hyperpolarizing voltage steps from −60.5 mV to potentials between −40.5 mV and −120.5 mV for 1 s in 5 mV step increment and then to −100.5 mV for 0.5 s to elicit the tail current to determine the voltage dependence of I_h_ activation. Current traces are representative for the dorsal, the intermediate and the ventral part of the MSO. **(C)** I-V relationships of steady-state (red arrow in **B**) I_h_ density for ventral (*n* = 15), intermediate (*n* = 12) and dorsal (*n* = 18) neurons emphasize that I_h_ density amplitudes are smallest in dorsal neurons and largest in ventral neurons **(C1)**. I_h_ density amplitudes for a voltage step to −110.5 mV **(C2)**. **(D)** Weighted activation time constants at −110.5 mV **(D1)**. The weighted activation time constants are voltage dependent and largest in the dorsal part of the MSO **(D2)**. **(E)** The voltage-dependence of I_h_ activation was measured from the tail current 20 ms after the end of the voltage steps (red arrow) **(E1)**. Values were fitted with a Boltzmann function to obtain the half-maximal activation voltage. In dorsal neurons the I_h_ activation curve is shifted to more negative voltages **(E2)**. Half-maximal activation voltage was measured in each experiment and averaged **(E3)**. Black symbols: dorsal neurons; gray symbols: intermediate neurons; white symbols: ventral neurons. ^**^P < 0.01, ^***^P < 0.001, single-factor ANOVA test followed by a Scheffe's *post-hoc* test.

Neuronal processing in the auditory system is tonotopically organized such that frequencies are orderly represented across most auditory nuclei. In the MSO, low frequency sounds are supposed to be encoded in the dorsal part of the MSO and higher frequency sounds are presumably represented in the ventral part (Guinan et al., 1972). In general, best frequencies of MSO neurons are lower compared to neurons in the LSO, but can occasionally still be above 2 kHz (Pecka et al., 2008). Here, we investigated in a brain slice preparation of P18 gerbils the biophysical properties of MSO neurons along this putative tonotopic axis. The MSO was subdivided into three regions, a ventral region, which we refer to as high-frequency (HF), a dorsal region, which we refer to as low-frequency (LF) and an intermediate middle-frequency (MF) region. MSO neurons were identified on the basis of their bipolar shape and their arrangement in a parasagittal plane. In some experiments, 100 μM Alexa-488 was included in the pipette solution to verify the visually determined location of the neurons along the dorsoventral axis (Figure 1A). The properties of I_h_ between the regions were analyzed using voltage-clamp experiments. In all cells hyperpolarizing voltage pulses triggered slowly activating, large inward currents. I_h_ amplitude was 57% larger in ventral (presumably HF) neurons compared with dorsal (presumably LF) neurons [at −110.5 mV: ventral: −3006 ± 165 pA; intermediated: −2388 ± 123 pA; dorsal: −1910 ± 278 pA; ANOVA: *F*_(2, 42)_ = 6.60, *P* = 0.003; Figure 1B]. Also, I_h_ density was significantly larger in the ventral part of the MSO compared with the dorsal and intermediate part [at −110.5 mV: ventral: −127.7 ± 5.3 pA/pF; intermediate: −85.1 ± 5.4 pA/pF; dorsal: −73.4 ± 9.3 pA/pF; ANOVA: *F*_(2, 42)_ = 14.86, *P* < 0.001; Figure 1C]. Dorsal neurons not only exhibited the smallest I_h_ amplitude but also I_h_ that activated slowest. The calculated weighted activation time constant was two-fold larger in dorsal neurons compared with ventral neurons [at −110.5 mV: ventral: 95.6 ± 7.0 ms; intermediate: 117.9 ± 9.2 ms; dorsal: 191.3 ± 28.1 ms; ANOVA: *F*_(2, 42)_ = 6.62, *P* = 0.003; Figure 1D1]. For all three regions, the weighted activation time constants were voltage-dependent with τ_weighted_ = 72 ± 5 ms at −120.5 mV increasing to τ_weighted_ = 215 ± 17 ms at −90.5 mV in the ventral part of the MSO (Student's paired *t*-test: *P* < 0.001) and with τ_weighted_ = 152 ± 22 ms at −120.5 mV increasing to τ_weighted_ = 367 ± 40 ms at −90.5 mV in the dorsal part (Student's paired *t*-test: *P* < 0.001) (Figure 1D2). Analyzing the amplitude of the tail current revealed that I_h_ voltage dependence was negatively shifted in dorsal neurons compared with ventral and intermediate neurons (Figure 1E). Consequently, the half-maximal activation voltage was most negative in dorsal neurons [ventral: −79 ± 1 mV; intermediate: −76 ± 2 mV; dorsal: −87 ± 2 mV; ANOVA: *F*_(2, 42)_ = 13.51, *P* < 0.001; Figure 1E]. On average, our measurements are in line with recently published data (Khurana et al., 2012).

Taken together, we observed a large difference in I_h_ properties between the ventral and the dorsal part of the MSO. Dorsal neurons exhibited smaller I_h_ amplitude, slower activation kinetics and more negative half-maximal activation voltage as compared to ventral neurons.

### cAMP modulation of I_*h*_ differs along the dorsoventral axis

**Figure 2:**
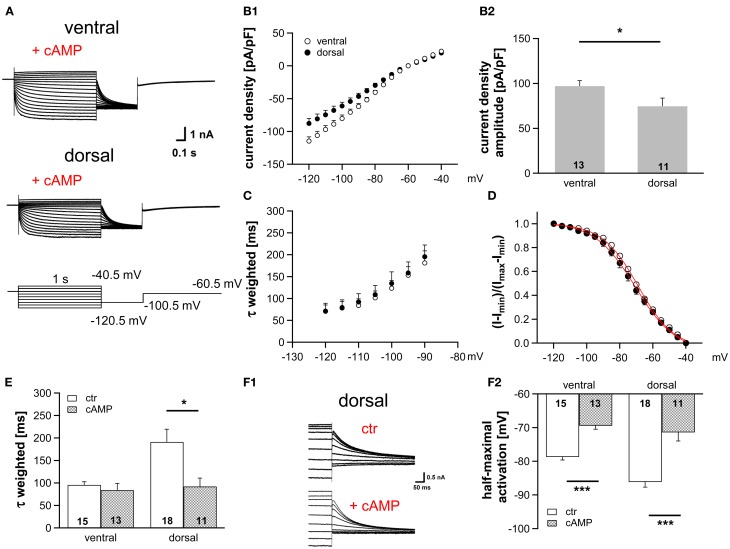
**Modulation of I_h_ by cAMP differs along the dorsoventral axis. (A)** Current responses to depolarizing and hyperpolarizing voltage steps were recorded from MSO neurons in the ventral and dorsal part of the MSO with 25 μM cAMP in the pipette solution. **(B)** I_h_ density gradient persists in the presence of cAMP as illustrated by the current-voltage relationships for ventral (*n* = 13) and dorsal (*n* = 11) neurons **(B1)** and their I_h_ density amplitudes for a −110.5 mV voltage step **(B2)**. **(C)** The weighted activation time constants and **(D)**, the voltage dependence of I_h_ activation overlap in the presence of cAMP. Comparison of **(E)** the weighted activation time constants and **(F)** the half-maximal activation voltages in the absence and presence of 25 μM cAMP reveals that dorsal neurons are more sensitive to cAMP than ventral neurons **(F2)**. **(F1)** In the upper panel, tail currents were elicited using standard pipette solution. In the lower panel, a different, dorsal neuron is illustrated using standard pipette solution supplemented with 25 μM cAMP. Black symbols: dorsal neurons; white symbols: ventral neurons. ^*^P < 0.05, ^***^P < 0.001, two-tailed, unpaired *t*-test.

HCN channel properties depend largely on the intracellular concentration of cAMP. The extent by which cAMP is able to regulate the gating of HCN channels is determined by the HCN subunits (Wahl-Schott and Biel, 2009). HCN1, which is less sensitive to cAMP, is the main subunit in MSO neurons (Koch et al., 2004; Khurana et al., 2012). Nevertheless, cAMP modulates the gating of HCN channels in the MSO probably due to a co-assembly of HCN1 and HCN4 to heteromeric HCN channels (Khurana et al., 2012). To test whether a cAMP-dependent modulation underlies the differences in I_h_ properties across the dorsoventral axis, we included 25 μM cAMP in the pipette solution, which induces maximal cAMP modulation (Ludwig et al., 1998). As expected, I_h_ density amplitude was still significantly larger in ventral neurons compared with dorsal neurons (at −110.5 mV: ventral: −99.9 ± 6.6 pA/pF; dorsal: −74.6 ± 6.8 pA/pF; Student's unpaired *t*-test, *P* = 0.014; Figures 2A,B). Moreover, cAMP accelerated the activation kinetics (Figures 2C,E) and positively shifted the activation curves in the two regions such that the activation curves overlapped for all neurons (Figures 2D,F), with the largest shift observed in dorsal neurons. Here, τ_weighted_ decreased more than two-fold from 191.3 ± 28.1 ms (*n* = 18) to 92.1 ± 18.8 ms (*n* = 11) at 110.5 mV (Student's unpaired *t*-test: *P* = 0.012, Figure 2E) and half-maximal activation voltage increased by 15 mV from −87 ± 2 mV (*n* = 18) to −72 ± 3 mV (*n* = 11) (Student's unpaired *t*-test: *P* < 0.001). In ventral neurons, no shift of τ_weighted_ was observed and half-maximal activation voltage was only shifted by about 9 mV (from −79 ± 1 mV (*n* = 15) to −70 ± 1 mV (*n* = 13); Student's unpaired *t*-test: *P* < 0.001; Figure 2F2). Hence, in the presence of saturating concentrations of cAMP the I_h_ activation kinetics and the dependence of I_h_ activation are similar whereas the dorsoventral difference of I_h_ amplitude persists. We, therefore, assume that the spatial arrangement of I_h_ density originates from differences in HCN channel density, whereas distinct basal intracellular cAMP levels cause the dorsoventral organization of the half-maximal activation voltage and the activation time constants.

### I_h_ differences affect membrane properties

**Figure 3:**
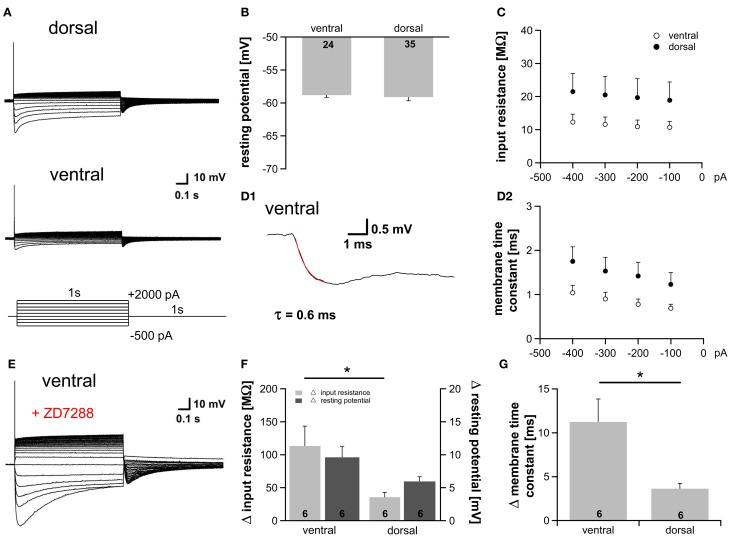
**Gradient of I_h_ affects membrane properties. (A)** MSO neurons in the dorsal and in the ventral part of the MSO fire a single spike at the onset of depolarizing current injections and exhibit a voltage sag during hyperpolarizing current steps. **(B)** Resting membrane potential, **(C)** peak input resistance, and **(D)** membrane time constant are not significantly different between ventral (*n* = 18) and dorsal (*n* = 25) neurons **(D2)**. The membrane time constant was fitted by a single-exponential function as shown in **(D1)**. **(E)** Blocking I_h_ with 20 μM ZD7288, a specific I_h_ blocker, hyperpolarizes the cell, increases the input resistance and the membrane time constant. Same ventral neuron as in **(A)** but after treatment with 20 μM ZD7288. The differences in **(F)** the input resistance (light-gray bars), the resting potential (dark-gray bars) and **(G)** the membrane time constant before and after application of 20 μM ZD7288 varies between ventral and dorsal neurons. The effects are more pronounced in ventral neurons. Black symbols: dorsal neurons; white symbols: ventral neurons. ^*^P < 0.05, two-tailed unpaired *t*-test.

At rest a fraction of HCN channels is open in the dorsal part (~9%) as well as in the ventral part (~15%) of the MSO (Figure 1E2). This is in accordance with studies showing that I_h_ plays a critical role in determining the membrane properties in auditory brainstem neurons (Golding et al., 1995; Adam et al., 2001; Koch and Grothe, 2003; Golding and Oertel, 2012). To test whether the observed differences in I_h_ result in diverse membrane properties we applied depolarizing as well as hyperpolarizing current injections and recorded the voltage responses from 59 neurons. As previously reported, depolarization of the cells elicited a single action potential at the onset of the current injection, whereas hyperpolarization induced a depolarizing voltage sag, which can be attributed to the activation of HCN channels (Figure 3A, Magnusson et al., 2005; Scott et al., 2005). Despite the different open probability of HCN channels at rest, the resting potential was nearly identical in all parts of the MSO (ventral: −58.8 ± 0.3 mV; dorsal: −59.1 ± 0.6 mV; Student's unpaired *t-test*: *P* = 0.692; Figure 3B) indicating compensatory gradient of outward currents. The peak input resistance and the membrane time constant did not differ significantly between the frequency regions, however, both showed clear trends. Ventral neurons tended to exhibit the lowest input resistance (at −100 pA: ventral: 10.7 ± 1.8 MΩ; dorsal: 18.9 ± 5.5 MΩ; Student's unpaired *t*-test: *P* = 0.240; Figure 3C). The membrane time constants were determined by fitting a single exponential function to the voltage traces (Figure 3D1). Ventral neurons tended to display the smallest membrane time constant (at −100 pA: ventral: 0.69 ± 0.09 ms; dorsal: 1.23 ± 0.27 ms; Student's unpaired *t*-test: *P* = 0.108; Figure 3D2). To solidify the observed trends, we repeated the experiments under bath application of 20 μM ZD7288, which selectively inhibits HCN channels. In all neurons, irrespective of their location along the dorsoventral axis, HCN channel blockade hyperpolarized the membrane potential and increased the input resistance and the membrane time constant (Figure 3E). This difference in input resistance and membrane time constant between control condition and HCN channel blockade varied significantly between dorsal and ventral neurons (Figures 3F,G). Thus, the fractional contribution of I_h_ is significantly different between dorsal and ventral neurons. The effects of 20 μM ZD7288 were more pronounced in the ventral part of the MSO (Figures 3F,G) demonstrating that I_h_ contribution to the membrane properties is larger in ventral neurons, and confirming that the distinct membrane properties along the dorsoventral axis can be attributed to the observed differences in I_h_.

### Integration of simulated inhibitory inputs varies along the dorsoventral axis

**Figure 4:**
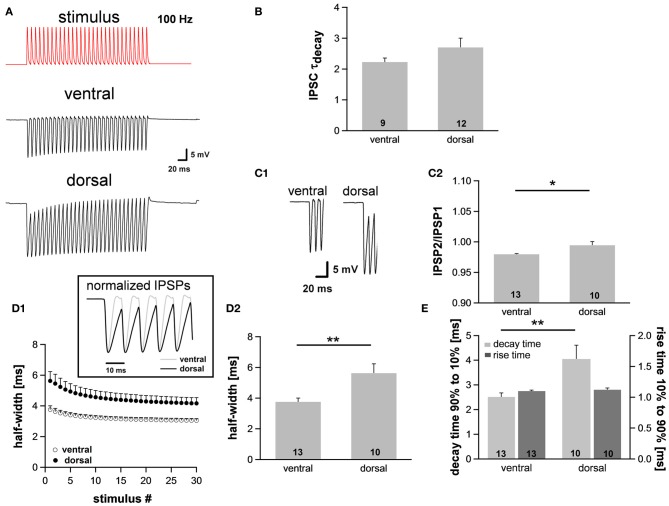
**The integration of synaptic inputs varies along the dorsoventral axis. (A)** Representative voltage traces to simulated IPSC trains (100 Hz). **(B)** The time course of IPSCs does not vary along the dorsoventral axis as indicated by the decay time. IPSCs were evoked by stimulating the slice medial or lateral the MSO with a stimulation electrode. **(C)** Dorsal neurons exhibit the largest IPSP amplitude **(C1)**, and IPSP summation is increased in dorsal neurons **(C2)**. **(D)** IPSP time course changes along the dorsoventral axis as depicted in the inset. The half-width of IPSPs is largest in dorsal neurons and the time course accelerates during stimulation with IPSC trains **(D1)**. Half-width for the first IPSP **(D2)**. **(E)** There is no difference between the rise times (10–90%) of the first IPSP (dark-gray bars) but the mean values for the decay time (90–10%) of the last IPSP (light-gray bars) are larger in dorsal neurons compared to ventral neurons. Black symbols: dorsal neurons; white symbols: ventral neurons. ^*^P < 0.05, ^**^P < 0.01, two-tailed paired or unpaired *t*-test, as appropriate.

Assuming the MSO receives inputs that are phase-locked to the fine structure of a sound, the temporal summation of IPSP should vary between the regions, being most prominent in ventral neurons that presumably receive HF inputs and least in dorsal neurons that presumably receive LF inputs. This summation would lead to a stronger hyperpolarization in ventral neurons and thereby reduce their excitability. In this case the observed dorsoventral difference of I_h_, which activates upon hyperpolarization, would compensate for the putatively increased hyperpolarization. To test our hypothesis, we simulated inhibitory inputs at 100 Hz and recorded the voltage responses from neurons in the dorsal and ventral part of the MSO. The simulated inhibitory conductance, which was injected into MSO neurons, was based upon recorded IPSCs (decay time: ~1.5 ms, 10–90% rise time: ~0.9 ms, amplitude: ~2 nA). We also confirmed that decay times of IPSCs did not differ significantly between the regions (ventral: 2.2 ± 0.1 ms, *n* = 9; dorsal: 2.7 ± 0.3 ms, *n* = 12; Student's unpaired *t*-test: *P* = 0.215; Figure 4B). These results are in line with data by Magnusson et al. (2005) showing that the IPSCs decay with time constants of around 1.5–3 ms in P18 gerbils (Magnusson et al., 2005).

As expected, the membrane potential response to the simulated IPSC trains varied as a function of the neuron's location along the dorsoventral axis (Figure 4A). The amplitude of the evoked IPSPs was significantly larger in neurons of the dorsal part compared with neurons of the ventral part (ventral: 18.3 ± 0.8 mV, *n* = 13; dorsal: 21.1 ± 0.9 mV, *n* = 10; Student's unpaired *t*-test: *P* = 0.035; Figure 4C1), also indicative for a larger input resistance in dorsal cells. Moreover, ventral neurons showed less summation than dorsal neurons (IPSP2/IPSP1: ventral: 0.980 ± 0.001, *n* = 13; dorsal: 0.995 ± 0.006, *n* = 10; Student's unpaired *t*-test: *P* = 0.011; Figure 4C2). To facilitate comparison of the time course, IPSPs were amplitude-normalized (Figure 4D1, inset) illustrating that the time course of IPSPs changed along the dorsoventral axis. The half-width of the IPSPs was largest in the dorsal part of the MSO and became smaller in the ventral part (ventral: 3.76 ± 0.25 ms, *n* = 13; dorsal: 5.63 ± 0.60 ms, *n* = 10; Student's unpaired *t*-test: *P* = 0.005; Figure 4D). There was no difference in 10–90% rise time of the first IPSP between the frequency regions (ventral: 1.10 ± 0.01 ms; dorsal: 1.12 ± 0.03 ms; Student's unpaired *t*-test: *P* = 0.450; Figure 4E), but the 90–10% decay time of the last IPSP was smallest in the ventral part (ventral: 2.51 ± 0.16 ms, *n* = 13; dorsal: 4.05 ± 0.56 ms, *n* = 10; Student's unpaired *t*-test: *P* = 0.008; Figure 4E). Taken together, the time course of the IPSPs is faster in ventral neurons than in dorsal neurons. We speculate that these effects can be attributed to the dorsoventral organization of I_h_ as HCN channels are the main channel subtypes that open upon hyperpolarization.

### Dorsoventral organization is preserved in more mature animals

**Figure 5:**
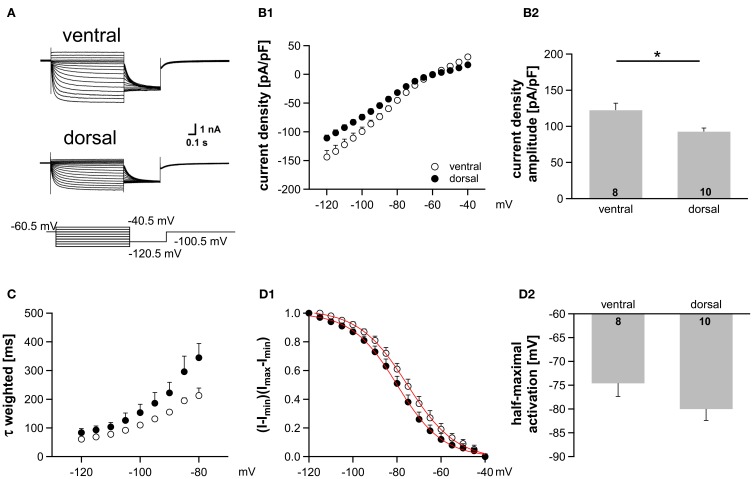
**I_h_ gradient persists in more mature animals (P22). (A)** Pharmacologically isolated I_h_ current traces were elicited by depolarizing and hyperpolarizing voltage steps from −60.5 mV to potentials between −40.5 mV and −120.5 mV (5 mV step increment). Current traces are representative for the dorsal and the ventral part of the MSO. **(B)** I-V relationships of steady-state I_h_ density for ventral (*n* = 8) and dorsal (*n* = 10) neurons emphasize that I_h_ density amplitudes are smallest in dorsal neurons and largest in ventral neurons **(B1)**. I_h_ density amplitudes for a voltage step to −110.5 mV **(B2)**. **(C)** The weighted activation time constants are voltage dependent and largest in the dorsal part of the MSO. **(D)** The voltage-dependence of I_h_ activation was measured from the tail current. In dorsal neurons the I_h_ activation curve is shifted to more negative voltages **(D1)**. Half-maximal activation voltage was measured in each experiment and averaged **(D2)**. Black symbols: dorsal neurons; white symbols: ventral neurons. ^*^P < 0.05, two-tailed unpaired *t*-test.

There is evidence that HCN channels in the superior olivary complex undergo drastic developmental changes during the first three postnatal weeks (Leao et al., 2006; Hassfurth et al., 2009; Khurana et al., 2012). To rule out that these developmental refinements have implications on the observed dorsoventral organization, we repeated experiments at P22, which is more at the end of this developmental period. Compared with P18, P22 MSO neurons exhibited slightly increased I_h_ amplitudes, the activation curves were shifted to more positive half-maximal activation voltages and the activation kinetics were accelerated (Table 1). Nevertheless, I_h_ still varied systematically along the dorsoventral axis, such that ventral neurons exhibited significantly larger I_h_ amplitudes than dorsal neurons (at −110.5 mV: ventral: −3304 ± 1.5 pA, *n* = 8; dorsal: −2560 ± 238 pA, *n* = 9; Student's unpaired *t*-test: *P* = 0.019; Figure 5A). Accordingly, I_h_ density also varied significantly (at −110.5 mV: ventral; −122.4 ± 9.6 pA/pF, *n* = 8; dorsal: −92.7 ± 5.0 pA/pF, *n* = 9; Student's unpaired *t*-test: *P* = 0.012; Figure 5B). Also, the activation kinetics and the half-maximal activation voltage differed between the ventral part and the dorsal part of the MSO (τ_weighted_ at −110.5 mV: ventral: 78.0 ± 3.6 ms, *n* = 8; dorsal: 109.6 ± 18.7 ms, *n* = 9; Student's unpaired *t*-test: *P* = 0.138; Figure 5C; *V*_0.5_: ventral: −76 ± 3 mV, *n* = 8; dorsal: −81 ± 2 mV, *n* = 9; Student's unpaired *t*-test: *P* = 0.160; Figure 5D). In P22 animals, I_h_ still was organized along the dorsoventral axis. However, these differences between ventral and dorsal neurons were less pronounced as compared to P18 gerbils.

**Table 1:** **Summary of HCN channel properties, membrane properties and synaptic properties of dorsal and ventral neurons for P18 and P22**.

	**P18**			**P22**		
	**Ventral**	**Dorsal**	***P***	**Ventral**	**Dorsal**	***P***
**HCN CHANNEL PROPERTIES (AT −110.5 mV)**						
Current [pA]	−3006 ± 165 (15)	−1909 ± 277 (18)	^**^	−3303 ± 134 (8)	−2559 ± 238 (9)	^*^
Current density [pA/pF]	−127.7 ± 5.3 (15)	−73.4 ± 9.3 (18)	^***^	122.4 ± 4.9 (6)	92.7 ± 5.0 (9)	^*^
Half-maximal activation voltage [mV]	−79 ± 1 (15)	−87 ± 2 (18)	^**^	−76 ± 3 (8)	−81 ± 2 (9)	n.s.
τ_weighted_ [ms]	95.6 ± 7.0 (15)	191.3 ± 28.1 (18)	^**^	78.0 ± 3.6 (8)	109.6 ± 18.7 (9)	n.s.
**MEMBRANE PROPERTIES (AT −100 pA)**						
Resting potential [mV]	−58.8 ± 0.3 (24)	−59.1 ± 0.6 (35)	n.s.	−60.0 ± 0.7 (10)	−60.1 ± 0.9 (12)	n.s.
Input resistance [MΩ]	10.7 ± 1.8 (24)	18.9 ± 5.5 (35)	n.s.	3.7 ± 7.0 (10)	24.0 ± 6.4 (12)	^*^
Membrane time constant [ms]	0.69 ± 0.09 (24)	1.23 ± 0.27 (35)	n.s.	0.45 ± 0.07 (10)	1.64 ± 0.45 (12)	^*^
**SYNAPTIC PROPERTIES**						
IPSP half-width [ms]	3.76 ± 0.25 (13)	5.63 ± 0.60 (10)	^**^	2.72 ± 0.06 (10)	4.29 ± 0.51 (13)	^*^
Rise time 10–90% [ms]	1.10 ± 0.01 (13)	1.12 ± 0.03 (10)	n.s.	1.14 ± 0.03 (10)	1.37 ± 0.07 (13)	^*^
Decay time 90–10% [ms]	2.51 ± 0.16 (13)	4.05 ± 0.56 (10)	^**^	1.84 ± 0.08 (10)	3.08 ± 0.39 (13)	^**^

Values are mean ± SEM. The level of significance between ventral and dorsal was determined by using Student's unpaired t-test except for HCN channel properties of P18, where Scheffe's post-hoc test was employed following a single-factor ANOVA test (

* *P < 0.05*,

** *P < 0.01*,

*** *P < 0.001*,

*n.s. not significant). The n-values for each group are stated in brackets*.

**Figure 6:**
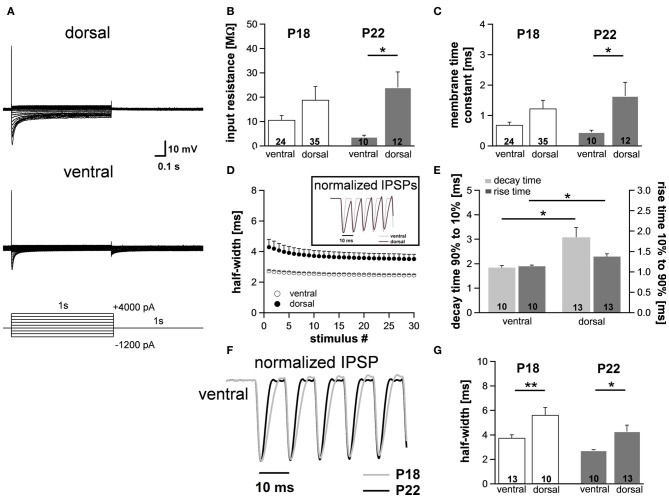
**Differences in membrane properties are more evident in mature animals. (A)** MSO neurons in the dorsal and in the ventral part of the MSO fire a single spike at the onset of depolarizing current injections and exhibit a voltage sag during hyperpolarizing current steps. In particular, in ventral neurons the voltage sag only is obvious for strong hyperpolarizing current injections due to the large resting conductance of I_h_. **(B)** Peak input resistance and **(C)** membrane time constant vary significantly between ventral (*n* = 10) and dorsal (*n* = 12) neurons. Differences in the input resistance and in the membrane time constant between ventral and dorsal neurons are significantly larger in P22 animals compared with P18 animals. **(D)** IPSP time course changes along the dorsoventral axis as depicted in the inset. The half-width of IPSPs is largest in dorsal neurons and the time course accelerates during stimulation with IPSC trains. **(E)** The mean values for the rise time (10–90%) of the first IPSP (dark-gray bars) and the decay time (90–10%) of the last IPSP (light-gray bars) are larger in dorsal neurons compared to ventral neurons. **(F)** IPSP time course is accelerated in P22 compared to P18. Representative normalized IPSP trains for the ventral part of the MSO. **(G)** Half-widths for the first IPSPs are decreased for P22 in both the ventral and the dorsal part of the MSO. Black symbols: dorsal neurons; white symbols: ventral neurons. ^*^P < 0.05, ^**^P < 0.01, two-tailed unpaired *t*-test.

To assess to what extent these subtle changes in HCN properties between P18 and P22 neurons affect the neurons' membrane properties, we also measured voltage changes in response to current injections in P22 animals. During depolarization neurons in the ventral part of the MSO fired a single spike at the beginning of the current injection (Figure 6A). In most ventral neurons, only for strong hyperpolarizing current injections the depolarizing voltage sag was obvious which is due to the extremely large I_h_. This is also reflected in the very low input resistance (at −100 pA: ventral: 3.7 ± 0.7 MΩ, *n* = 10; dorsal: 24.0 ± 6.4 MΩ, *n* = 12; Student's unpaired *t*-test: *P* = 0.016; Figure 6B) and in the very small time constant of ventral neurons (at −100 pA: ventral: 0.45 ± 0.07 ms, *n* = 10; dorsal: 1.64 ± 0.45, *n* = 12; Student's unpaired *t*-test: *P* = 0.047; Figure 6C). Compared with P18 gerbils, the differences in the membrane time constant and in the input resistance between ventral and dorsal neurons were larger which resulted in significant differences along the dorsoventral axis (Figures 6B,C; Table 1).

We evaluated the integration of inhibitory postsynaptic inputs by injecting currents with stimulus amplitudes adjusted to evoke physiological IPSPs of similar sizes (−8.1 ± 0.3 mV in the ventral part, *n* = 10, and −8.4 ± 0.3 mV in the dorsal part of the MSO, *n* = 12; Student's unpaired *t*-test: *P* = 0.493). Similar to P18, the voltage response to the simulated IPSC trains varied along the dorsoventral axis. The half-width of the first IPSP (ventral: 2.73 ± 0.06 ms, *n* = 10; dorsal: 4.29 ± 0.51 ms, *n* = 13; Student's unpaired *t*-test: *P* = 0.014; Figure 6D), the 10–90% rise time of the first IPSP (ventral: 1.14 ± 0.03 ms, *n* = 10; dorsal: 1.37 ± 0.07 ms, *n* = 6; Student's unpaired *t*-test: *P* = 0.007; Figure 6E) as well as the 90–10% decay time of the last IPSP (ventral: 1.84 ± 0.08 ms, *n* = 10; dorsal: 3.08 ± 0.39 ms, *n* = 13; *P* = 0.013; Figure 6E) were largest in the dorsal part of the MSO and became smaller in the ventral part. By comparing the time course of P18 and P22 neurons (Figure 6F, example for ventral neurons) we can demonstrate that consistent with an increase in I_h_ the half-width of the IPSPs is decreased in P22 neurons (Figure 6G). This emphasizes our hypothesis that I_h_ accelerates the time course of the IPSP and thereby decreases the temporal summation of IPSP. In addition, I_h_ compensates the summated hyperpolarization induced by the temporal summation of HF inhibitory inputs.

Taken together, these data provide evidence that also in mature animals the integration of synaptic inputs varies as a function of the neuron's location along the dorsoventral axis and that a tonotopic organization of I_h_ may at least partially account for the observed gradient in synaptic integration.

### Tonotopic organization of I_*h*_ accounts for the dorsoventral differences in synaptic integration

**Figure 7:**
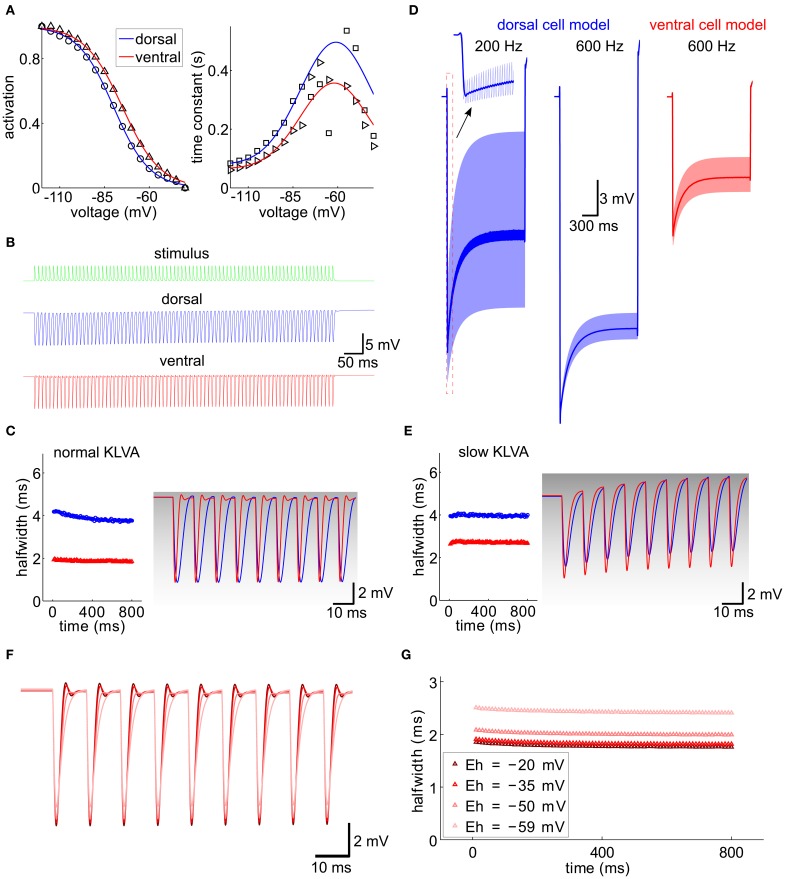
**Integration of IPSG trains, computational model. (A)** Activation curve and time constant of I_h_ from Figure 5 (symbols) were fitted for the ventral and dorsal population (solid lines). **(B)** A 100 Hz IPSG train (green) was applied to the cell models representative for the dorsal (blue) and ventral (red) population (see Materials and Methods). The individual inhibitory conductance was set to 20.5 nS and 90 nS for the dorsal and for the ventral cell models to obtain similar voltage amplitudes. **(C)** Half-width of the resulting IPSPs from **(B)**. The first nine IPSPs were magnified on the right and overlaid with the respective K_LVA_ steady-state activation (gray levels). **(D)** Voltage responses (light colors) for different stimulus frequencies (as indicated) and model cells (colors). The dark traces were generated by low-pass filtering the voltage trace with a second-order Butterworth low-pass filter with cut-off frequency of 100 Hz. **(E)** Same as **(C)** for a K_LVA_ model with 100-fold slowed down activation and inactivation time constants. Conductances for I_h_ and I_K-LVA_ were adjusted to match the input resistances and equilibrium potential of the cells in **(C)**. **(F)** IPSP trains for different reversal potentials of I_h_ (color code see **G**). **(G)** Half-widths of the IPSP trains from **(F)**.

To gain further mechanistic understanding and to assess the functional consequences of the dorsoventral I_h_ gradient in a computational model of a MSO cell, we first fitted activation profiles and channel time constants of I_h_ (from Figure 5) as described in the Materials and Methods section (Figure 7A). In addition to I_h_, the model also included a low-voltage activated potassium current I_K−LVA_ to counteract I_h_ induced depolarization (Svirskis et al., 2002; see Materials and Methods). The peak conductances of I_h_ and I_K−LVA_ were used as free parameters to adjust the neuron models to a given input resistance and resting potential. Whereas the former was taken to be 3.77 MΩ for ventral (putative HF) neurons and 23.94 MΩ for dorsal (putative LF) neurons, the latter was assumed identical (−60 mV) in both populations.

We first validated our models by reproducing the current clamp experiments from Figure 6F (Figures 7B,C). Applying a stimulus of 100 Hz, we measured the half-width of the inhibitory potentials for cell models with both dorsal and ventral characteristics. The simulated IPSP half-widths are in very good agreement with the experimental data.

Following the idea that the dorsoventral differences parallel the tonotopic axis, we simulated the response of the model neuron to periodic inhibitory inputs with different frequencies (Figure 7D). The kinetics of the individual IPSGs was modeled to fit those measured experimentally (Couchman et al., 2010 and Materials and Methods). The decay constant τ = 1.6 ms of these IPSGs is so slow that there is temporal summation of the IPSPs, which produces a significant hyperpolarizing voltage offset (dark lines in Figure 7D). This offset increased for higher stimulation frequencies (200 Hz vs. 600 Hz in the example of Figure 7D). The increase of the hyperpolarizing voltage offset for HF inputs can, however, be mitigated, if we assume that only the ventral neurons process HF inputs: In those neurons this offset is smaller because of the lower input resistance that results from larger I_h_ and I_K−LVA_ conductances (Figure 7D).

The kinetics of I_h_ are much slower than the time constants that are typical for fast auditory processing. Therefore, I_h_ is generally assumed not to be suited to directly interact with neuronal processing of sound information on a fast time scale. However, the interplay between I_h_ and I_K−LVA_ may play an important role in temporal sharpening of the PSPs (Khurana et al., 2011). In contrast to I_h_, the I_K−LVA_ does possess fast kinetics and thus has been proposed to contribute to fast temporal processing of MSO neurons in several studies (Svirskis et al., 2002; Jercog et al., 2010). To specifically evaluate the interaction between I_h_ and I_K−LVA_ channel kinetics in the present context of IPSG trains, we also simulated a model in which the I_K−LVA_ kinetics had been slowed down such that the kinetics were comparable to the I_h_ kinetics, while leaving the input resistance unchanged. Comparing both models (fast I_K−LVA_ kinetics, Figure 7C and slow I_K−LVA_ kinetics, Figure 7E) we found a clear effect on temporal precision as measured by an increase in IPSP half-width. This increase was stronger for the model of the ventral MSO neuron (Figure 7E), i.e., a putative HF processing neuron, although the input resistance was the same for both I_K−LVA_ kinetics. This shows that for high frequencies neurons with large I_h_ the temporal precision of the hyperpolarizing IPSPs is considerably enhanced by the active properties of the fast K_LVA_ channels, whereas for low frequency neurons this temporal integration is mostly explained by the differences in input resistance. Mechanistically, the I_h_-dependent sharpening of IPSPs can be understood as follows: During the hyperpolarizing flank of the IPSPs the I_K−LVA_ channels – which are open at rest – close very rapidly and thereby effectively set a new equilibrium potential of the whole cell at a depolarized level close to the reversal of I_h_. The resulting huge driving force massively speeds up the depolarizing flank of the IPSP and thereby accounts for the temporal sharpening. As the membrane potential approaches the old equilibrium potential, the K_LVA_ channels quickly open again and they restore the original equilibrium potential with only little overshoot as witnessed by the small amplitude of the voltage fluctuation after the IPSPs in Figure 7C. To test the above hypothesis, we conducted simulations with different reversal potentials of I_h_, As expected, a reduction of the driving force broadened the IPSPs (Figures 7F,G).

In summary, we conclude that fast K_LVA_ channels in interaction with I_h_ may predominantly sharpen the IPSPs (particularly in HF neurons with large I_h_), whereas I_h_ in MSO neurons alone balances out the hyperpolarizing voltage offset induced by the temporal summation of phase-locked inhibitory synaptic currents.

## Discussion

In the present study we demonstrate that I_h_ amplitude systematically varies along the dorsoventral axis of the MSO, being largest in ventral neurons and smallest in dorsal neurons. Consistent with this dorsoventral organization of membrane properties, the integration of inhibitory inputs systematically varies as a function of the neuron's location in both experiments and the model indicating that MSO neurons are tuned differentially along the presumed tonotopic axis. Tonotopic gradients of I_h_ have been previously observed in auditory brainstem nuclei. For example, in the lateral superior olive (LSO) I_h_ is larger in the LF region of the nucleus compared to the HF region (Hassfurth et al., 2009). This opposite gradient might be due to the fact that in general LSO processes much higher input frequencies compared to MSO neurons (Sanes et al., 1989; Tolnai et al., 2008). The I_h_ gradient is also opposite in the nucleus laminaris (NL) (Yamada et al., 2005), the bird's MSO analogue ITD processing stage. Whether this difference is due to the diverse function of inhibitory inputs in mammals and birds or to different ITD processing strategies in the two animal classes is not clear. However, these results suggest that tuning of biophysical membrane properties through differential expression of HCN channels along the tonotopic axis in general optimizes the processing of different inputs frequencies (Kuba et al., 2005; Slee et al., 2010).

In mammals, I_h_ (or HCN) channels can derive from four different genes (HCN1-4) and assemble into homo- or heterotetramers with distinct electrophysiological properties in terms of their activation kinetics, their activation dependence, and their sensitivity to cAMP. In contrast, single-channel conductance is very similar for the different HCN isoforms (Brandt et al., 2009) and maximal I_h_ amplitude depends only very little on intracellular modulators (Ludwig et al., 1998; Wahl-Schott and Biel, 2009). This suggests that the observed dorsoventral gradient of I_h_ density in the MSO most likely relies on differences in the number of HCN channels and is independent of subunit variation. Our experiments also show that I_h_ activation kinetics accelerates and half-maximal activation voltage increases from the dorsal to the ventral part of the MSO. A distinct distribution of the isoforms along the dorsoventral axis might provide an explanation for the differences in biophysical properties of I_h_ (Yamada et al., 2005). MSO neurons mainly express HCN1 and HCN4 subunits which both possess distinct physiological properties (Khurana et al., 2012). Among all different HCN subunits, HCN4 possesses the slowest kinetics and the most negative half-maximal activation voltage, whereas HCN1 possesses the fastest kinetics and the most positive half-maximal activation voltage (Santoro et al., 2000; Moosmang et al., 2001). Thus, an increased contribution of HCN1 towards ventral neurons could result in faster activation kinetics and more positive half-maximal activation voltage. Conversely, our data suggest that different basal levels of intracellular cAMP cause the observed dorsoventral gradient in I_h_ properties. The gating of HCN channels in MSO neurons is very sensitive to cAMP, since most likely HCN1 and HCN4 isoforms co-assemble to form fast-activating but cAMP-sensitive HCN heteromers (Khurana et al., 2012). We show that dialyzing neurons with a saturating cAMP concentration resulted in nearly identical activation kinetics and half-maximal activation voltages in all MSO neurons. This opens the possibility that differential activation or expression of receptors that modulate intracellular cAMP levels could modify I_h_ properties along the presumed tonotopic axis of the MSO (Yamada et al., 2005) and regulate processing of various input frequencies in an activity dependent manner.

Functionally, I_h_ strongly influences basic membrane properties such as resting potential, input resistance and membrane time constant of neurons. These properties determine cellular excitability and synaptic integration. More specifically, I_h_ depolarizes the resting potential toward spike threshold, decreases the membrane time constant and lowers the input resistance at and below the resting potential, when the membrane potential is hyperpolarized in response to inhibitory inputs. Consistent with this idea we found that ventral neurons had a lower input resistance and a faster membrane time constant than dorsal neurons. In addition, postsynaptic integration of inhibitory inputs differed dependent on I_h_ amplitude and properties.

But what are the functional implications for the diversity of I_h_ of different neuron types for information processing in a small network? This and the relation to I_h_ has been extensively studied in both the entorhinal cortex and the hippocampus where I_h_ properties and HCN channels are as well distributed along a dorsoventral gradient (Garden et al., 2008; Giocomo and Hasselmo, 2008; Marcelin et al., 2012a,b). In these structures, I_h_ has been hypothesized to contribute to the observed gradient in grid field spacing in the entorhinal cortex (Giocomo et al., 2011; Hussaini et al., 2011). This mostly relates to the fact that I_h_ accelerates resonance frequency in those neurons. In these neurons I_h_ also tunes the membrane properties to the slow oscillatory activity of the inputs they receive, which is crucial for the specific function of these neurons. In auditory brainstem neurons and especially in the MSO, input frequencies (up to 1.5 kHz) are a magnitude higher than the activation and deactivation kinetics of I_h_ and thus an active contribution to temporal processing is unlikely.

One possible explanation why I_h_ distribution is tonotopically organized is suggested by our model and our experimental data. Neurons in the MSO not only receive two precisely timed excitatory but also two prominent inhibitory inputs from the medial and lateral nucleus of the trapezoid body (Grothe et al., 2010), which are phase-locked to the fine-structure of the sound. Due to the relatively slow time constants of the inhibitory inputs (Magnusson et al., 2005; Couchman et al., 2010), the inhibition summates and strongly hyperpolarizes the neuron. Since I_h_ is rapidly activated during hyperpolarization we propose that I_h_ reduces the integration of synaptic inputs during periods of prolonged hyperpolarization. Indeed, both our experimental data and our model show that I_h_ decreases the temporal summation of IPSPs by gradually activating and thereby opposing the summated hyperpolarization induced by the temporal summation of HF inhibitory inputs. Functionally such a hyperpolarizing offset is problematic, since it effectively increases the spike threshold and thereby strongly reduces or even completely prohibits neuronal spiking in response to these input frequencies. The additional I_h_ activated in the ventral MSO region prevents this excessive hyperpolarization and keeps the neurons in an operating regime for binaural coincidence detection. In MSO neurons both I_h_ and I_K−LVA_ are open at rest (Khurana et al., 2011) and both contribute to the extremely low membrane time constants. The balance between the hyperpolarizing I_K−LVA_ and the depolarizing I_h_ determines the resting potential and together lowers the membrane time constants in both the hyperpolarizing and the depolarizing range. This decrease in time constant also in the depolarizing range would then improve coincidence detection of inputs thereby optimizing ITD analysis in these neurons. In addition, a higher expression level of I_h_ also indirectly enhances I_K−LVA_-induced sharpening of inhibitory synaptic potentials by modulating the speed of depolarization via the driving force of I_h_ (Figures 7C,E). Conversely, two recent studies suggest that increasing I_h_ in the MSO and the NL, the bird's analogue structure of the MSO, sharpens the time window for coincidence detection also of excitatory inputs (Yamada et al., 2005; Khurana et al., 2012). This all implies that MSO neurons that respond best to higher frequency sounds and have thus larger I_h_ should have sharper time windows for ITD detection compared to neurons responding best to low frequency sounds. This phenomenon can indeed be observed for ITD functions of MSO neurons that are tuned to different best frequencies (Yin and Chan, 1990; Brand et al., 2002).

### Conflict of interest statement

The authors declare that the research was conducted in the absence of any commercial or financial relationships that could be construed as a potential conflict of interest.
